# The Psychosis Recent Onset GRoningen Survey (PROGR-S): Defining Dimensions and Improving Outcomes in Early Psychosis

**DOI:** 10.1371/journal.pone.0113521

**Published:** 2014-11-20

**Authors:** Edith J. Liemburg, Stynke Castelein, Frank van Es, Anne Neeltje Scholte-Stalenhoef, Gerard van de Willige, Henderikus Smid, Ellen Visser, Henderikus Knegtering, Richard Bruggeman

**Affiliations:** 1 Rob Giel Research Center, University of Groningen, University Medical Center Groningen, Groningen, The Netherlands; 2 NeuroImaging Center, Department of Neuroscience, University of Groningen, University Medical Center Groningen, Groningen, The Netherlands; 3 Lentis Research, Lentis Mental Healthcare Center, Groningen, The Netherlands; Chiba University Center for Forensic Mental Health, Japan

## Abstract

Psychotic disorders are among the most complex medical conditions. Longitudinal cohort studies may offer further insight into determinants of functional outcome after a psychotic episode. This paper describes the Psychosis Recent Onset in GRoningen Survey (PROGR-S) that currently contains data on 1076 early-episode patients with psychosis, including symptoms, personality, cognition, life events and other outcome determinants. Our goal in this report is to give an overview of PROGR-S, as a point of reference for future publications on the effect of cognition, personality and psychosocial functioning on outcomes. PROGR-S contains an extensive, diagnostic battery including anamnesis, biography, socio-demographic characteristics, clinical status, drug use, neuropsychological assessment, personality questionnaires, and physical status tests. Extensive follow-up data is available on psychopathology, physical condition, medication use, and care consumption. Sample characteristics were determined and related to existing literature. PROGR-S (period 1997–2009, n = 718) included the majority of the expected referrals in the catchment area. The average age was 27 (SD = 8.6) and two-thirds were male. The average IQ was lower than that in the healthy control group. The majority had been diagnosed with a psychotic spectrum disorder. A substantial number of the patients had depressive symptoms (479/718, 78%) and current cannabis or alcohol use (465/718, 75%). The level of community functioning was moderate, i.e. most patients were not in a relationship and were unemployed. The PROGR-S database contains a valuable cohort to study a range of aspects related to symptomatic and functional outcomes of recent onset psychosis, which may play a role in the treatment of this complex and disabling disorder. Results reported here show interesting starting points for future research. Thus, we aim to investigate long-term outcomes on the basis of cognition, personality, negative symptoms and physical health. Ultimately, we hope that this paper will contribute improving the health of patients with psychotic disorders.

## Introduction

Psychotic disorders are among the most disabling and expensive medical conditions with a lifetime prevalence of almost 3% [1]. Diagnostic assessment is complex due to the multifactorial etiology and heterogeneous course of such disorders [2]. Whereas some patients may recover, others experience an unfavorable course of illness [3], [4]. The reasons for this heterogeneous course are still unclear, but intervention in the early stages of the illness may be an important determinant for functional recovery [5]–[7]. Longitudinal cohort studies may offer insight into determinants of functional outcome after a psychotic episode. The goal of this paper is to give an overview of such determinants using the Psychosis Recent Onset in GRoningen Survey (PROGR-S) for the first time. This paper should also serve as a point of reference for future publications.

Besides genetic predisposition, life events may determine whether an individual develops psychosis. First episode patients have a higher incidence of traumatic life events [6], which are predictive for the development of psychosis [8]. Personality characteristics and coping skills, e.g. for stress management also influence the risk of developing psychosis [9]–[11]. Social relationships may help to reduce stress and inhibit the conceptualization of delusional ideas [12], but individuals with psychosis often experience problems maintaining peer relationships [13]. A satisfying living situation and occupation also reduce the risk of developing a psychosis [12].

Lower educational achievements and IQ levels have also been associated with increased risk for psychosis [6], [13] and poor social outcomes [14], [15]. These factors may be indicative of cognitive impairments, which are key symptoms of psychotic disorders [16], [17]. However, a substantial variation in impairment of cognitive functioning is observed across psychotic patients [18]–[20] and 25% of patients exhibit cognitive performance within normal limits [21], [22]. Thus, cognition could be a predictor of outcome, along with positive and negative symptoms [23].

Treatment of psychosis should start as early as possible, since a longer duration of untreated psychosis (DUP) may predict poorer outcomes [24]. Antipsychotics are a first and important step in treatment, but functional recovery is often poor despite initial symptom reduction [25], [26]. On the other hand, continuation of antipsychotic treatment may prevent relapses [27], at the cost of serious side effects. Owing to these complex considerations, the optimal treatment strategy for an individual patient is difficult to determine on the basis of current knowledge.

Psychotic patients often have an unhealthy lifestyle (smoking, sedentary, unhealthy food intake) [28], [29]. Combined with antipsychotic treatment, this lifestyle is a risk factor for metabolic syndrome, obesity, movement disorders, liver and kidney dysfunction, diabetes and cardiovascular problems [30]–[33]. Moreover, psychotic patients often use substances that may exacerbate psychosis, such as alcohol, cannabis, amphetamines, hallucinogens, and sedatives [34]. Routine screening for metabolic problems may help to reduce the risk of comorbid physical disorders [30], [35]–[37].

Other cohort studies have already focused on first-episode samples, but often on the basis of a limited diagnostic range, poor definition of the catchment area, unstructured information on environmental and contextual factors, lack of information on interventions and neuropsychological function, small sample sizes, no control group, and limited follow-up data [6], [7], [24], [38].

This paper provides an overview of the research setting of the Psychosis Recent Onset in GRoningen Survey (PROGR-S) from the Northern Netherlands. PROGR-S is designed to collect data on symptoms, cognition, personality, life events, drug abuse, psychological and physical status, and other determinants that may influence functional outcome in recent-onset psychotic patients [5]–[7], [38]. To date, PROGR-S contains 1076 participants with a recent onset psychosis, and growing, and a matched healthy control group. PROGR-S can be linked to databases with detailed follow-up information on outcome, including a database with annual measurements of physical and psychological health status, social and occupational functioning and quality of life (For an overview of this database: Bartels et al., in prep.), and two databases on daily care consumption and daily medication use.

## Methods

### Study sample

The diagnostic protocol for PROGR-S was established in 1997 for all inhabitants (550,000) in the Groningen province in the Netherlands who were referred to a psychiatric institution with a suspected recent onset psychotic episode (<2 years) or evaluated for a recurrent psychotic episode not diagnosed as such before. There was no exclusion based on age, diagnoses, substance abuse, or ethnicity. The database currently contains 1076 patients. A sample of 718 validated cases was analyzed for the current report (1997–2009). Between 1997 and 2009, an average of 62 patients were included each year in the PROGR-S database. Certain patients from this period were excluded after referral for the following reasons: no consent for scientific research (n = 5), no show/stopped (n = 13), non-native language (n = 5), second opinion or previously included (n = 5), moved or referred (n = 5), unknown reason (n = 4).

All study procedures were carried out in accordance with the declaration of Helsinki. All data was primarily collected for clinical purposes. Participants gave oral and written informed consent after procedures had been fully explained on the use of the data in our research database, as approved by the Institutional Review Board (IRB). All procedures were in accordance with local and international rules as confirmed by the local ethical committee of the University Medical Center of Groningen. The medical ethical committee of the University Medical Center Groningen declared that their approval was not required, as data were collected for diagnostic purposes, no interventions outside standard care were performed, and data were anonymized for research purposes.

Clinical interviews took place at the clinic at the time of admission. Psychological testing and interviews on clinical background information were carried out after two months, because the first florid psychotic symptoms have often remitted by that time, and would likely interact with test results. All procedures were conducted in Dutch in accordance with standardized protocols. For neuropsychological assessments and personality questionnaires, patients had to be native Dutch speakers. The results of these tests are standardized to the Dutch population. The PROGR-S-protocol takes 7–9 hours to complete, divided over two sessions.

### Measurements

PROGR-S serves as a baseline measurement after a first psychosis. For a large number of patients, a yearly follow-up measurement is also avaiable (Pharmacotherapy Outcome and Monitoring Survey; PHAMOUS; Bartels et al., in prep.). Moreover, there is a daily registration of care consumption by the Psychiatric Case Registry Northern Netherlands (PCRNN). For an overview of all measures, see File S1. The PROGR-S-protocol included the following assessments:

Anamnesis (medical history) and biography: obtained from the patient and, if possible, together with close relations (mostly parents) for hetero-anamnesis and information on childhood development. Anamnesis included nature, severity, and consequences of symptoms for daily functioning, along with the use of medication, recreational drugs, and family anamnesis (history of illness in family). The biography focused on signs of an early developmental disorder, childhood and current psychological traumas (such as abuse, neglect, and personal loss), and the highest level of social and intellectual functioning reached, including living situation and occupation.

Clinical and diagnostic data: The Positive and Negative Syndrome Scale (PANSS): a semi-structured interview on symptom severity of psychotic disorders including three subscales, Positive symptoms, Negative Symptoms and General psychopathology [39]; Montgomery-Asberg Depression Rating Scale (MADRS ): a semi-structured interview on the severity of depressive symptoms that may be more sensitive to treatment effects than other depression interviews [40], [41]; Schedules for Clinical Assessment in Neuropsychiatry (SCAN ): a comprehensive psychiatric diagnostic interview (Diagnostic and Statistical Manual of Mental Disorders; DSM-IV) and severity on the basis of an algorithm [42]; Global Assessment of Functioning (GAF): a DSM-IV rating by the clinician indicating the level of social, occupational, and psychological functioning of the individual [43]. The Camberwell Assessment of Needs (CAN) measures whether the clinical and social needs of people with severe mental illnesses are being met [44].

Functioning measurements: Groningen Social Disabilities Schedule (GSDS): a schematic interview on disabilities in social functioning in the domains of self-care, household activities and relations with close family, extended family and partner, societal integration, relationships with friends, work, and daily activities [45]; Psychological assessment including most neurocognitive domains of the Measurement and Treatment Research to Improve Cognition in Schizophrenia) Consensus Cognitive Battery (MATRICS)-Consensus [46], except social cognition: the Stroop Test [47], [48], Trail Making Test [49], California Verbal Learning Test, Dutch edition [50], Continuous Performance Test [51], Finger Tapping Test [52], and the Digit Symbol Substitution, Block Design, Arithmetic and Information subtest of the Wechsler Adult Intelligence Scale (WAIS) III [53], administered in a fixed order within approximately two hours.

Personality measures: Neuroticism-Extroversion-Openness - Five-Factor Inventory (NEO-FFI): a self-report personality questionnaire that includes five important domains of personality, namely Neuroticism, Extraversion, Openness, Agreeableness, and Conscientiousness [54] -Dutch version [55]; Utrecht Coping List (UCL): a self-report questionnaire measuring a range of coping strategies, including Problem solving, Distraction, Avoidance, Social support, Passive coping, Emotional expression, and Comforting [56].

Physical health status: Physical examination includes blood pressure, heart rate, height and weight. If necessary, additional tests can be performed; Laboratory tests include testing for general health conditions, anemia, liver function, signs of diabetes, risk factors for heart and vascular disease, kidney function, pituitary function, and syphilis.

Training and instruction of test psychologists for the neurocognitive battery were conducted on site in order to ensure uniform testing. Psychiatrists were trained by the Groningen World Health Organization (WHO) Training Center to administer the SCAN diagnostic interview (see below). Training of the research nurses for PANSS and GSDS (see below) was provided at investigator meetings, supplemented by written training materials. Training for the PANSS and GSDS included rating a videotaped interview, followed by discussion and review of ratings in accordance with strict guidelines (e.g. PANSS score should not deviate more than one point per item). Booster meetings were organized annually, to maintain inter-rater reliability.

### Control sample

A healthy control sample (n = 70) was selected based on random sampling from the community through the local municipal administration. Selected controls (n = 1000) received an information letter informing them about the study and inviting them to participate. In total, 93 controls responded to the invitation and 70 were eligible for inclusion in the study. Five individuals had to be excluded and 18 declined to participate or were no shows. Controls were matched with patients on age, gender and highest educational level. A male/female ratio similar to the patient sample was included, and because onset of psychosis generally occurs early in life, we selected healthy subjects between 18 and 50 years old. Exclusion criteria for controls included a psychiatric or neurological history or a first-degree family member with a psychiatric diagnosis (defined as absence of any lifetime psychiatric symptoms assessed with the screening questions of the SCAN-interview) [42]. Subjects were excluded if they had an excessive alcohol intake (21 units per week for males and 15 for females; according to Dutch government guidelines), more than one unit of cannabis per week or any hard drugs. Participants were instructed to abstain from cannabis and alcohol for 24 hours prior to testing. The research protocol for controls included the cognitive test-battery, NEO-FFI, UCL, and demographic data including age, gender, educational achievements and occupation.

### Research data management

Certified medical staff performed data collection. Patient information for clinical purposes was stored in the local mental health care information system and in paper medical records. Research assistants entered data for the research database from the medical records using the Statistical Package for the Social Sciences (SPSS 20; IBM Inc. New York, USA). Personal data and research data could not be linked directly. Researchers using the database received an anonymized version. The PROGR-S database is too comprehensive and valuable to be made publicly available without any restrictions. In accordance with the PLOS ONE data sharing policy, data from the PROGR-S cohort is available by contacting Edith Liemburg (e.j.liemburg@umcg.nl). Persons requesting data should fill in a short data-request form indicating their research question and aims, desired variables, and a short description of the analysis plan. After approval by the PROGR-S steering committee, a custom-made database with the requested data will be provided.

For an overview of the sample, socio-demographic and clinical data are presented in this paper. Socio-demographic data are shown for patients and healthy controls: education level (elementary school (1) up to university (8) [57], ethnicity (native or not), occupation, living situation and IQ (based on WAIS III [53]). Clinical data on patients is also included: DSM-IV diagnosis (grouped according to Bromet et al. [58], GAF score, PANSS score, MADRs score and categorization of severity of depression according to Hermann et al. [59], number of psychotic episodes, duration of untreated psychosis (DUP), lifetime drug/alcohol use, and use of antipsychotics (haloperidol equivalents according to Andreasen et al. [60].

Due to non-normal distribution of the data, a Mann Whitney U test had to be used to compare patients with healthy controls on age, IQ and education level, and a Chi-square test for independence to test for differences in gender distribution. Other characteristics, such as occupation, were only compared visually between both groups.

## Results

The PROGR-S database currently includes 1076 patients. In this paper, we included a validated set of 718 patients (1997–2009). Table 1 gives an overview of the socio-demographic characteristics of both patients and healthy controls (n = 70). The average age is 27 years (SD = 8.6) and 73% of the sample is male. Patients and healthy controls were of a similar age (*Z* = 0.88, *p* = 0.38). Although onset of psychosis is often later in females than males, the age distribution for males and females was similar in patients and matched healthy controls, except that female controls had a non-significant larger age range (*Z* = 1.0, *p* = 0.32) (25%, 50%, 75%; male patients: 21, 25, 31 years; male controls: 22, 25, 29; female patients: 22, 26, 33; female controls: 21, 29, 44). Despite specific selection of males at later stages of the study, the gender ratio was significantly different in each group (*χ* ^2^ = 8.7, *p* = 0.003). Patients also had a lower level of the highest level of education reached (*Z* = 8.0, *p*<0.005) and a lower IQ (*Z* = 7.7, *p*<0.0005). Only one third of patients had a paid job and almost half were unemployed, whereas only one person in the control group was unemployed. Moreover, 50% of the control sample were students versus only 16% of patients.

**Table 1:** Overview of demographical and clinical characteristics of the patients and controls in PROGR-S, the last column give the p-value of the comparison between both groups.

		Patients	Controls	p-value
		*mean (SD; range)* */number (%)*	*mean (SD; range)* */number (%)*	
**Age**		27.7 (8.6; 16–69)	28.8 (9.3; 18–49)	0.38
**Gender**	*Male*	525 (73.0)	39 (55.7)	0.003
	*Female*	193 (27.0)	31 (44.3)	
**Education**	*Elementary school*	4 (0.6)	1 (1.4)	<0.0005
**highest level**	*Secondary school*	122 (16.9)	1 (1.4)	
**reached** 1	*High school*	58 (8.1)	3 (4.3)	
	*Vocational education*	245 (34.1)	4 (5.7)	
	*University*	259 (36.1)	61 (87.2)	
	*Other*	30 (4.2)	0	
**IQ** 2		93.3 (14.3; 54–138)	110.7 (14.7; 75–140)	<0.0005
**Ethnicity**	*Native*	594 (82.7)	97.1	
	*Non-native*	124 (17.3)	2.9	
**Occupation**	*Unemployed*	309 (43.0)	1 (1.4)	
	*Paid job*	219 (30.5)	37 (52.9)	
	*Voluntary job*	39 (5.4)		
	*Student*	114 (15.9)	31 (44.3)	
	*Other*	7 (1.0)	0	
**Living situation**	*Married*	35 (4.9)	9 (12.9)	
	*Living alone*	353 (49.2)	14 (20.0)	
	*Living with parents*	242 (33.7)	2 (2.9)	
	*Partner (not married)*	50 (7.0)	26 (37.1)	
	*Mental health institute*	22 (3.1)	0	
	*Other*	16 (2.2)	0	

1 According to Verhage, 1984 [57].

2 Based on the WAIS III [53].

Table 2 shows the clinical characteristics of the patient sample. As patients could be referred for any type of disorder that may include psychotic symptoms, some patients had a primary diagnosis outside the psychotic spectrum. Moreover, due to the recent onset of psychosis, a definitive diagnosis could often not be established at that point, resulting in a high incidence of differential and comorbid diagnoses. The other primary diagnoses in Table 2 included the following: cannabis abuse/dependence (n = 8), other substance abuse/dependence (n = 3), autistic/developmental problems (n = 5), somatoform disorder (n = 2), Tourette's syndrome (n = 1), dissociative disorder (n = 2), identity problems (n = 2), and others (n = 5) = . It is worth noting that these patients had a diagnosis on the psychotic spectrum as differential or comorbid diagnosis. The average level of functioning according to the GAF was moderate (mean = 54.4, SD = 13.8). Although all patients were treated for a first episode of psychosis, 14.4% reported similar psychotic symptoms in the past, and were therefore reported here as second or third episode psychosis. The DUP was shorter than three months for 36% (for the known cases; 175 out of 479). The average severity of symptoms during the PANSS-interview was mild to moderate, i.e. an average PANSS score per item of 2–3 (mild-moderate). A substantial number of individuals reported depressive symptoms and current substance use, mainly cannabis or alcohol. 80% of the patients were already receiving antipsychotic treatment at the time of inclusion in PROGR-S, with an average dosage of 6 mg (SD = 3.8) of haloperidol equivalents.

**Table 2:** Clinical characteristics of participants in PROGR-S.

		mean	SD	range	%
**Diagnosis** 1	*No diagnosis*				0.7
	*Schizophrenia*				42.3
	*Substance induced psychosis*				4.2
	*Psychotic disorder, other*				20.2
	*Schizophreniform disorder*				9.0
	*Schizoaffective disorder*				4.5
	*Delusional disorder*				3.2
	*Bipolar disorder*				4.8
	*Affective disorders, other*				6.0
	*Other diagnoses*				5.1
**GAF** 2		54.4	13.8	16–99	
**DUP**	*<1 month*				10.3
	*1*–*2 month*				6.7
	*2*–*3 month*				7.3
	*>3 month*				42.3
	*Unknown*				33.3
**Number of psychotic episodes**	*One*				74.6
	*Two*				8.8
	*Three*				5.5
	Unknown				11.1
**PANSS Positive subscale**		12.6	4.8	7–32	
**PANSS Negative subscale**		14.3	6.0	7–41	
**PANSS General subscale**		29.7	8.1	16–64	
**MADRS** 3		13.2	8.9	0–40	
	*Normal/symptoms absent*				25.1
	*Mild depression*				44.7
	*Moderate depression*				20.2
	*Severe depression*				1.8
	Unknown				8.2
**Lifetime**	*Cannabis*				61.7
	*Alcohol*				44.5
	*Other substances*				2.3
**Present state (3 months)**	*Cannabis*				29.5
	*Alcohol*				12.7
	*Other substances*				0.7
**Use of antipsychotics (dose & % using)**	*Medication naive*				19.1
**Use of common antipsychotics (dose & % using)**	*Risperidone*	3.2	1.5	1–10	23.7
	*Olanzapine*	12.3	5.6	2–30	28.0
	*Quetiapine*	463.7	244.9	50–1000	7.9
	*Clozapine*	360.4	135.3	127–750	5.2
	*Aripiprazole*	13.1	6.0	7.5–30	3.6
	*Other oral antipsychotics*				7.9
**Haloperidol equivalents** 4		6.3	3.8		

1 According to SCAN-interview (Giel and Nienhuis, 1996) [42].

2 1 = severe dysfunction, 100 = optimal functioning.

3 Categorized according to Herrmann et al. (1998) [59].

4 According to Andreassen et al., 2010 [60].

## Discussion

This article presents the objectives, recruitment strategies, assessment methods, and sample characteristics of the PROGR-S database. Many factors influence outcomes for patients with psychosis [58]. PROGR-S will enable us to study these factors in more detail. The descriptive overview of the present paper [61] sets the stage for future PROGR-S-based reports.

Between 1997 and 2009, an average of 62 patients were included every year in the PROGR-S database. The province of Groningen has a population of 550,000. On the basis of an incidence of psychosis of between 10 and 20 per 100 000 persons in the Netherlands [62], we estimate that between 55 and 110 persons within the province will develop psychosis every year. This indicates that PROGR-S has a relatively good catchment and therefore a representative geographic cohort. In the following paragraphs, relevant sample characteristics are discussed and comparisons are drawn with other studies.

The socio-demographic characteristics of our sample fit the general picture for patients with a psychotic illness [24], [38], [63]–[65]. Most patients in the sample were aged between 20 and 30 years, and the male - female ratio was 2: 1 [66]. Moreover, most patients were unemployed and lived alone or with their parents. Healthy peers often had a job and higher educational achievements than patients. This may indicate that patients with a recent onset psychosis have less beneficial living conditions than their peers without a disorder. Social engagement and personality are important indicators for outcome in psychosis [67]–[69]. At present, research is underway on how personality and social participation influence outcomes for psychotic patients. Unfortunately, information on the living situation of healthy controls was not recorded. However, our database will be linked to socio-demographic data (including living situation) collected by the Dutch government, which is expected to provide interesting opportunities for future research.

Patients had an average IQ of 93, whereas the average IQ of the control group was 111. Notably, a relatively large proportion of the patient group had university level education, which can be explained by the fact that Groningen is a university town. It has been shown that neurocognitive deficits already existed before the first episode of psychosis, and that cognitive therapy may help to improve cognition, in contrast with medication [70], [71]. One of our goals will be to investigate whether first episode cognitive function predicts functional outcome later on.

Approximately 36% of the patients experienced a DUP shorter than three months and there was a wide variety in length. These findings are similar to those of other studies [24], [63], [65]. Unfortunately, a long DUP may result in an unfavorable outcome for a recent onset psychosis [72]. The wide variety in DUP we observed may be an interesting starting point to study the effect of treatment in early versus late stages of psychosis [73]. The GAF score was around 55 (optimal functioning  = 100), which seems to be in line with other first episode psychosis studies [24], [63], [64]. Combined with the mild to moderate PANSS scores [38], these results indicate that patients were not severely ill at the time the diagnostic interview was administered. This may be explained by the use of antipsychotics at the time of assessment. The majority of the patients were already taking antipsychotic medication. The average dose of 6 mg haloperidol equivalents is relatively high according to current indications [74]. However when data collection began, higher doses were common practice. Dosages declined over time in our sample, with an average of 6.4 in the first year and 5.6 in the last year. Some of the patients used multiple antipsychotics or relatively high doses of first-generation antipsychotics. Moreover, the haloperidol equivalents calculated [60] may be relatively high, e.g. for risperidone. It is worth noting that although minimal intervention with medication is often advised, recent studies show that discontinuation lead to a high risk of symptom reoccurrence [75]. Furthermore, a quarter of patients show persistent symptoms after treatment[76], and treatment response may be predicted based on baseline factors [77]. The follow-up databases contains detailed information on medication prescriptions and related health status of the patients, because improvement of treatment and physical health is our main research goal.

Three quarters of the patients in the PROGR-S database reported past or current use of cannabis or alcohol. This fits with earlier findings in other studies on psychotic patients [63], [78]. It has been shown that individuals with a psychotic disorder who use cannabis are more likely to develop psychotic symptoms than healthy controls [79], [80] and that cannabis may cause long-term cognitive impairments [81]. Scientific knowledge on the effect of alcohol abuse on psychosis is limited. We hope that our database will provide important information on the effect of cannabis, alcohol, and other drugs on the clinical course after a psychosis.

According to the MADRS, a high incidence of mild to moderate depression was present in the PROGR-S patient sample (n = 479/78%), which fits with earlier reported incidences of depression in psychotic patients [63], [82], including in our follow-up sample [83]. Given the major burden of depression on top of other symptoms of psychosis, this should be an important focus for future research.

The primary goal of PROGR-S is to collect data for diagnostic assessment; therefore the database may not be entirely suited for scientific purposes. However, the large naturalistic sample, covering more than 75% of patients with psychosis in the province of Groningen may outweigh this, as generalization to the population is desirable. Moreover, socio-demographic matching of our healthy control sample was limited, despite our efforts. We will extend the control group to achieve better matching.

Opportunities to link the information to other existing databases add to the value of PROGR-S. In this way, costs savings can be achieved as outcomes can be derived from existing initiatives.

In conclusion, the PROGR-S database is a valuable geographic cohort that can be used to study various aspects that may play a role in the treatment of psychosis. In future studies, we will examine hypotheses on the effect of neurocognitive capacities, psychosocial functioning, personality traits and coping styles on functional outcome. Ultimately, we hope to gain more insight into outcome of psychotic disorders that will contribute to health improvements in patients with psychotic disorders.

## Supporting Information

File S1 **Measures and inclusion in PROGR-S and the measures and number of linked cases in the follow-up databases.**
(DOCX)
