# Supplementary material for: The Psychosis Recent Onset GRoningen Survey (PROGR-S): Defining Dimensions and Improving Outcomes in Early Psychosis
Source: PLoS One. 2014 Nov 20;9(11):e113521. doi: 10.1371/journal.pone.0113521 (PMC4239096; doi:10.1371/journal.pone.0113521)
Supplement: File S1 — Measures and inclusion in PROGR-S and the measures and number of linked cases in the follow-up databases. (DOCX) [file pone.0113521.s001.docx]

**S1 Measures and inclusion in PROGR-S and the measures and number of linked cases in the follow-up databases**

|  | **PROGR-S** | **PHAMOUS** | **PCRNN** |
| --- | --- | --- | --- |
|  | *Baseline (1997-2009)* | *Yearly follow-up*  *(2006-2013)* | *Daily follow-up*  *(2000-2012)* |
|  | *(N = 718)* | *(N = 349 48.6%)* | *N = 718 (100%)* |
| **Demographical data** | Socio-demographic  (N=718, 100%) | Socio-demographic  (N=349, 49%) | Demographic (N=718, 100%) |
| **Diagnosis** | DSM IV (N=712, 99%) | DSM IV (N=349, 49%) | DSM IV (N=666, 92.8%) |
| **Psychopathology** | PANSS  (N=710, 99%) | PANSS (N=340,47%) |  |
|  | MADRS (N=659, 92%) | CDSS (N=206, 29%) |  |
|  | GAF (N=662, 92%) | GAF (N=316, 44%) |  |
| **Treatment history** | Onset symptoms & treatment  (N=485, 68%) | Onset symptoms & treatment (N=337, 47%) |  |
|  | Medication use (N=615, 86%) | Medication use  (N=348, 48%) |  |
|  | SRA (5 years) | SRA (N=282, 39%) |  |
| **Family history** | Psychiatric disorders N=614, 86%) |  |  |
|  | Somatic disorders (N=310, 43%) | Somatic disorders  (N=346, 48%) |  |
| **Life events** | Semi-structured biography  (N=607, 93%) |  |  |
| **Substance use** | Alcohol/cannabis/  hard drugs (self-report) (N=718, 100%) | Alcohol/cannabis/  hard drugs (self-report)  (N=348, 48%) | |
| **Physical health** | Physical examination  (n=220, 31%) | Physical examination (N=348, 48%) |  |
|  | Laboratory test  (N=586, 82%) | Laboratory test  (N=334, 47%) |  |
|  |  | Diet and exercise  (N=348, 48%) |  |
| **Cognition and personality** | Cognitive battery  (N=622, 87%) | |  |
|  | NEO-FFI  (N=587, 82%) |  |  |
|  | UCL (N=546, 76%) |  |  |
| **Psychosocial functioning** | Vocational functioning  (N=688, 96%) | Vocational functioning  (N=272, 38%) | |
|  | GSDS (N=562, 78%)  MANSA (2 years) | MANSA (N=343, 48%) |  |
|  | HoNOS (2 years) | HoNOS (N=345, 48%) |  |
| **Care consumption** |  | Care consumption  (self-report)  (N=349, 49%) | Medical file registration  (N=695, 96.8%) |
| **Care needs/satisfaction** | CAN (N=562, 78%) | CSQ (N=274, 38%) |  |

Abbreviations: PHAMOUS: Pharmacotherapy Monitoring and Outcome Survey; PCRNN: Psychiatric Case Registry Northern Netherlands; CDSS: Calgary Depression Rating Scale [1]; CSQ: Client Satisfaction Questionnaire [2]; MANSA: Manchester Short Assessment of Quality of Life [3]; HoNOS: Health of the Nation Outcome Scales [4]; SRA: Subject’s Response to Antipsychotics [5,6]

g

References

1. Addington D, Addington J, Schissel B (1990) A depression rating scale for schizophrenics. Schizophr Res 3: 247-251.

2. Larsen DL, Attkisson CC, Hargreaves WA, Nguyen TD (1979) Assessment of client/patient satisfaction: Development of a general scale. Eval Program Plann 2: 197-207.

3. Priebe S, Huxley P, Knight S, Evans S (1999) Application and results of the manchester short assessment of quality of life (MANSA). Int J Soc Psychiatry 45: 7-12.

4. Wing JK, Beevor AS, Curtis RH, Park SB, Hadden S, et al. (1998) Health of the nation outcome scales (HoNOS). research and development. Br J Psychiatry 172: 11-18.

5. Wolters HA, Knegtering H, Van den Bosch RJ, Wiersma D (2009) Effects and side effects of antipsychotic treatment in schizophrenia: Pros and cons of available self-rating scales. Schizophr Res 112: 114-118.

6. Lako IM, Bruggeman R, Liemburg EJ, van den Heuvel ER, Knegtering H, et al. (2013) A brief version of the subjects' response to antipsychotics questionnaire to evaluate treatment effects. Schizophr Res 147: 175-180.
